# Serum 25(OH)D is inversely associated with metabolic syndrome risk profile among urban middle-aged Chinese population

**DOI:** 10.1186/1475-2891-11-68

**Published:** 2012-09-09

**Authors:** Xiao Yin, Qiang Sun, Xiuping Zhang, Yong Lu, Chao Sun, Ying Cui, Shaolian Wang

**Affiliations:** 1 Department of Endocrinology and Metabolism, Shandong University Affiliated Jinan Central Hospital, Jinan, China; 2 Center for Health management and Policy, Shandong University, Jinan, 250012, China; 3 Experiment Diagnostic Center, Shandong University Affiliated Jinan Central Hospital, Jinan, China; 4 Healthcare Department, Shandong University Affiliated Jinan Central Hospital, Jinan, China

**Keywords:** Obesity, Vitamin D deficiency, Metabolic syndrome

## Abstract

**Background:**

Vitamin D deficiency is associated with a variety of chronic metabolic diseases. Limited evidence regarding vitamin D deficiency exists within the Chinese population. The present study aims to examine the association between serum vitamin D concentrations and cardiometabolic risk factors in the young and middle-aged, urban Chinese population

**Methods:**

The cross-sectional relationships between serum 25-hydroxyvitamin D [25(OH)D] concentrations and indices of adiposity and cardiometabolic risk factors (e.g., body mass index, waist circumference, fasting plasma glucose, etc.) were evaluated in 601 non-diabetic adults.

**Result:**

Vitamin D deficiency or insufficiency was present in 66% of the tested population, and serum 25(OH)D levels were lower in patients who were overweight/obese or suffered metabolic syndrome when compared to individuals of healthy weight without metabolic syndrome (24.08 ± 8.08 vs 31.70 ± 11.77 ng/ml, 21.52 ± 6.9 vs 31.74 ± 10.21 ng/ml respectively). 25(OH)D was inversely associated with waist circumference, fasting glucose, fasting insulin, triglycerides and LDL-cholesterol, and it was positively associated with HDL-cholesterol in a multivariable-adjusted regression model.

**Conclusion:**

Vitamin D deficiency is common in the young and middle-aged, urban Chinese population, with high prevalence in overweight/obese individuals and patients with metabolic syndrome. Low vitamin D concentration was associated with indices of adiposity and cardiometabolic risk factors. Further studies are warranted to elucidate the cause-effect relation between vitamin D status, obesity and related metabolic disorders.

**Trial registration:**

Current Controlled Trials (ISRCTN21527585)

## Introduction

Altered vitamin D homeostasis is associated with increased risk of developing obesity [1-4], hypertension [5,6], glucose intolerance and metabolic syndrome [7]. Additionally, hypovitaminosis D has been reported as a risk factor for increased cardiovascular events [8] and was independently associated with all-cause mortality based on analysis of the Third National Health and Nutrition Examination Survey (NHANES III) data.

Vitamin D status, which was assessed by serum 25-hydroxyvitamin D [25(OH)D] concentrations, differs among ethnic groups, with African-Americans, Hispanics and Asians, having a greater prevalence of hypovitaminosis D [9,10]. Lower vitamin D levels among these groups may be explained in part by darker skin pigmentation [11]. The relationship between hypovitaminosis D and metabolic traits, such as insulin resistance, appear to vary among different ethnicities. NHANES III data [12] showed an inverse association between vitamin D status and insulin resistance in non-Hispanic whites and Mexican Americans, but the inverse relationship did not hold in African-Americans Americans. Limited information exists concerning the relationship between vitamin D status and metabolic risk factors among Asian populations. Because of the increased risk of vitamin D deficiency among the northern Chinese population due to their darker skin pigment and northern latitude, coupled with the increasing incidence of obesity and metabolic syndrome, we seek to determine if hypovitaminosis D is associated with metabolic risk factors in the Chinese population.

This study aims to 1) determine the cross-sectional association of vitamin D status with indices of adiposity and metabolic risk factors in a representative sample of urban, young and middle-aged, non-diabetic adults residing in northern China, and 2) evaluate the prevalence of subclinical vitamin D deficiency in this cohort.

## Methods

### Study sample

This study included 601 adults that received health examinations in Jinan Central Hospital. Inclusion criteria were as follows: 35–60 years old, living in Jinan (latitude 36.6) for more than 5 years, employed in an office setting, and > 13 years of education. Exclusion criteria included the following: use of vitamin D and calcium supplementation within 60 days of screening, current use of cigarettes (self-reported), alcohol abuse (defined as >14 drinks/week for men, >7 drinks/week for women), diagnosis of overt diabetes, cardiovascular disease or other systematic disease, use of medications that influence vitamin D, glucose, lipid profiles or blood pressure, or engaging in ≥20 min of strenuous physical activity or exercise that causes excessive breathing and sweating ≥ 1 time per week. This study was approved by the Jinan Central Hospital Ethics Committee and was performed in accordance with the declaration of Helsinki. All subjects provided written informed consent.

### Clinical assessment

Body mass index (BMI) was categorized as normal weight (<24 kg/m^2^) , overweight (24–28 kg/m^2^) and obese (>28 kg/m^2^), according to the criteria for Chinese individuals [13]. Metabolic syndrome was identified using the updated National Cholesterol Education Program Adult Treatment Panel III criteria for Asian Americans [14] and includes presentation of three or more ofthe following components: 1) waist circumference ≥90 cm for men or ≥80 cm for women; 2) triglyceride ≥1.7 mmol/L; 3) HDL cholesterol <1.03 mmol/L for men or <1.30 mmol/L for women; 4)blood pressure ≥130/85 mmHg; and 5) fasting glucose ≥5.6 mmol/L. Vitamin D nutritional status was based on 25(OH)D levels, which were assessed as “deficient”(<20 ng/ml), “insufficient”(20-30 ng/ml) or “sufficient”( >30 ng/ml) [15].

### Protocol

Anthropometric measures (weight, height, and waist circumference) and blood pressure were calculated on all participants. A fasting blood sample was collected in November and December for measurement of serum 25(OH)D, plasma glucose, insulin, triglyceride, LDL-cholesterol and HDL-cholesterol concentrations. The homeostasis model assessment of insulin resistance (HOMA-IR) was calculated from fasting insulin and glucose levels [16].

### Laboratory analyses

Serum samples were obtained in the morning after an overnight fast and frozen at −80°C. Serum 25(OH)D and insulin were measured by double antibody radioimmunoassay (DiaSorin, Stillwater, MN, and Linco Research, St. Charles, MO) with quality control materials provided by the manufacturer. The inter-assay coefficient of variation for 25(OH)D and insulin were 9.3% and 6.7% respectively. Plasma glucose, triglycerides and HDL cholesterol were measured by enzymatic colorimetric assay on a Bayer 2400 chemistry analyzer (Bayer Corporation). LDL cholesterol was calculated using the Friedwald equation. All of the intra- and inter-assay coefficients of variation were <10%.

### Statistical analyses

Descriptive characteristics for participants are expressed as median (interquartile range) for continuous variables with skewed distribution, means (SD) for continuous variables with normal distribution and percent for categorical variables. Some variables (body mass index, waist circumference, fasting insulin, homair, and HDL) were logarithmically transformed when analyzed. Comparisons of anthropometric and metabolic characteristics were made using unpaired t-test or rank-sum test between groups with and without vitamin D deficiency. Comparison of groups with different vitamin D statuses were made using one-way ANOVA analysis (for continuous variables with normal distribution) or rank transformation test (for categorical and continuous variables with skewed distribution).

Multivariable linear regression was performed in the total sample to examine the association of 25(OH)D (dependent variable) with the following clinical, anthropometric, and metabolic variables (independent variables) age, sex, BMI, waist circumference, fasting glucose, fasting insulin, HOMA-IR, triglycerides, HDL cholesterol, systolic blood pressure, and diastolic blood pressure. The association of each variable with 25(OH)D was initially examined with adjustment for age and sex. We then conducted multivariable regression analyses with stepwise forward selection to evaluate the independent association of 25(OH)D with each of the clinical, anthropometric, and metabolic variables listed above. A *P* value < 0.10 was the significance criterion for covariates to enter and remain in the regression model.

## Results

Clinical and biochemical characteristics of the 601 included subjects are shown in Table 1. The mean age of participants was 50 years. Approximately one fourth of the participants were female, and the mean 25(OH)D concentration was 26.91 ng/ml. 398 subjects (66%) had 25(OH)D concentrations of 30 ng/ml or less, and vitamin D deficiency (serum 25(OH)D concentration < 20 ng/ml) was present in 172 subjects (28.6%). 63% of the cohort were overweight/obese, and 44% of the tested population suffered from metabolic syndrome. Vitamin D insufficiency and deficiency were present in 76.2% and 88.3% in overweight/obese and patients with metabolic syndrome respectively.

**Table 1:** Characteristics of samples

	**Total (n = 601)**
Age (years)	49.36 ± 7.10
Female (%)	25.46%
Waist circumference (cm)	87(80–93)
BMI (kg/m^2^)	25.21(22.94-27.19)
Fasting plasma glucose (mmol/L)	5.2(5.6-5.9)
Fasting insulin (μIu/ml)	12.83(8.29- 19.53)
HOMA-IR	3.14 (2.05- 4.8)
Triglycerides (mmol/L)	1.58 (1.15- 2.25)
HDL cholesterol (mmol/L)	1.2(1.03-1.4)
LDL cholesterol (mmol/L)	3.32(2.86-3.84)
Systolic blood pressure (mmHg)	132(119–144)
Diastolic blood pressure (mmHg)	85(75–93)
25(OH)D (ng/ml)	26.91(10.41)

Data are median (interquartile range), mean (SD) or n(%).

We categorized the subjects using the referenced vitamin D nutritional status cutoff (as described in methods) into vitamin D deficiency, insufficiency and sufficiency groups which have no significant difference in age and sex (Table 2), this definition was based on the fact that the serum iPTH will rise significantly once the serum 25(OH)D level drops to less than 30 ng/ml [17], and those less than 20 ng/ml is likely to have clinically skeletal effects of vitamin D deficient state. The vitamin D deficiency group had significantly greater waist circumference, BMI, fasting plasma glucose, insulin, HOMA-IR, triglycerides, and diastolic blood pressure and lower HDL cholesterol compared to vitamin sufficiency and insufficiency groups. LDL cholesterol and systolic blood pressure were significantly lower in the vitamin D sufficiency group compared to the two other groups, but there was no significant difference between vitamin D deficiency and insufficiency groups. 15.2% individuals of the vitamin D sufficiency group suffered from metabolic syndrome, but for vitamin D insufficiency and deficiency groups, the incidence rate of metabolic syndrome was higher (48.6% and 72.6% respectively).

**Table 2:** Characteristics of samples according to vitamin D status

	**Vitamin D status**		
	**Sufficiency**	**Insufficiency**	**Deficiency**
n	203	226	172
Age (years)	47.98 ± 7.27	50.29 ± 6.40	49.77 ± 7.54
Female (%)	30	23	23
Waist circumference (cm)	80(76–86)	88(81–93)^*^	92(87.5-99)^*#^
BMI (kg/m^2^)	23.72(21.77-25.69)	25.21(23.75-27.12)^*^	26.63(24.79-29.07)^*#^
Fasting plasma glucose (mmol/L)	5.3(5.1-5.9)	5.6(5.3-5.9) ^*^	5.9(5.5-6.15) ^*#^
Fasting insulin (μIu/ml)	9.83(6.68-12.96)	13.75(9.68-18.57)^*^	18.13(11.18-28.63)^*#^
HOMA-IR	2.28(1.56-3.09)	3.41(2.44-4.73) ^*^	4.77(2.84-7.38) ^*#^
Triglycerides (mmol/L)	1.2(0.95-1.49)	1.71(1.21-2.2) ^*^	2.36(1.61-3.20) ^*#^
HDL cholesterol (mmol/L)	1.31(1.14-1.52)	1.18(1.02-1.37) ^*^	1.10(0.93-1.26) ^*#^
LDL cholesterol (mmol/L)	2.25(2.8-3.69)	3.29(2.86-3.72) ^*^	3.49(2.97-4.20) ^*^
Systolic blood pressure (mmHg)	125(115–137)	135(120–147) ^*^	136.5(126.5-150) ^*^
Diastolic blood pressure (mmHg)	80(71–88)	85(76–94) ^*^	90(80–96) ^*#^
25(OH)D (ng/ml)	38.36(8.09)	25.07(2.63) ^*^	15.81(3.06) ^*#^
Metabolic syndrome(%)	15.27	48.67	72.67

Waist circumference, BMI, Fasting insulin, HOMA-IR, HDL cholesterol, LDL cholesterol were log-transformed before analysis. * P < 0.05 compared to vitamin D sufficiency group; # P < 0.05 compared to vitamin D insufficiency group.

The participants were also divided into metabolic syndrome (MS) and non-metabolic syndrome (non-MS) groups according to the criterion described in methods. The 25(OH)D level in MS group (21.52 ± 6.9 ng/ml) was significantly lower compared to individuals without metabolic syndrome (31.74 ± 10.7 ng/ml, P < 0.05). The incidence rate of vitamin D deficiency and insufficiency was higher in MS group compared to non-MS group (88.3 % vs. 48.6%).

The associations of 25(OH)D with clinical and metabolic traits are shown in Table 3. 25(OH)D was negatively related to BMI after adjusted for age and sex. Significant inverse associations were noted between 25(OH)D and each of the remaining clinical and metabolic covariates, including waist circumference, fasting glucose, fasting insulin, HOMA-IR, triglycerides, LDL cholesterol and systolic blood pressure, after adjusted for age, sex and BMI. Additionally, serum 25(OH)D was positively associated with HDL cholesterol.

**Table 3:** Age and sex adjusted relations of serum 25(OH)D and metabolic covariates (n = 601)

	Coefficient(SE)*	***P***
Age	−0.24(0.05)	<0.0001
sex	−3.76(0.92)	<0.0001
BMI	−1.72(0.13)	<0.0001
Waist circumference	−0.63(0.06)	<0.0001
Fasting plasma glucose	−6.53(0.67)	<0.0001
Fasting insulin	−0.39(0.04)	<0.0001
HOMA-IR	−1.49(0.14)	<0.0001
Triglycerides	−2.51(0.27)	<0.0001
HDL cholesterol	8.67(1.43)	<0.0001
LDL cholesterol	−1.33(0.49)	0.0067
Systolic blood pressure	−0.06(0.02)	0.0162
Diastolic blood pressure	−0.07(0.03)	0.0455

*Coefficients represent change in 25(OH)D (ng/ml) for an increase in the value of the predictor variables shown (1-SD increase for continuous predictor variables).

Results of the stepwise multivariable regression model are shown in Table 4. Because of the larger variance observed for HOMA-IR and its collinear relationship with fasting insulin (r = 0.987, P < 0.0001), HOMA-IR was excluded from the stepwise multivariable regression model. Systolic and diastolic blood pressure did not meet significance requirements for entry into the model, and therefore, were not included (P = 0.83, P = 0.75 respectively). All of the remaining variables entered into and remained significant in the stepwise forward regression model, including age, sex, BMI, waist circumference, fasting glucose, fasting insulin, triglyceride, total cholesterol, HDL cholesterol and LDL cholesterol.

**Table 4:** Stepwise multivariable adjusted relationships of clinical and metabolic covariates with serum 25(OH)D

	Coefficient(SE)*	**Beta**	***P***
**All (N = 601)**			
Age	−0.24(0.05)	−0.164	<0.0001
Sex	−6.77(0.88)	−0.286	<0.0001
BMI	−0.56(0.16)	−0.160	0.0004
Waist circumference	−0.22(0.07)	−0.209	0.0015
Fasting plasma glucose	−4.63(0.61)	−0.239	<0.0001
Fasting insulin	−0.17(0.04)	−0.164	<0.0001
Triglycerides	−1.46(0.26)	−0.193	<0.0001
HDL cholesterol	4.50(1.27)	0.121	0.0004
LDL cholesterol	−1.20(0.41)	−0.089	0.0034
**Men (N = 448)**			
Age	−0.30(0.06)	−0.197	<0.0001
BMI	−0.59(0.18)	−0.164	0.001
Waist circumference	−0.28(0.075)	−0.233	<0.0001
Fasting plasma glucose	−3.87(0.71)	−0.16	<0.0001
Fasting insulin	−0.16(0.04)	−0.154	0.001
Triglycerides	−1.23(0.34)	−0.177	<0.0001
HDL cholesterol	4.28(1.61)	0.110	0.008
**Women (N = 153)**			
Fasting plasma glucose	−7.42(1.28)	−0.382	<0.0001
Fasting insulin	−0.21(0.09)	−0.188	0.025
Triglycerides	−3.38(1.35)	−0.228	0.014

*Coefficients represent change in 25(OH)D (ng/ml) for an increase in the value of the predictor variables shown (1-SD increase for continuous predictor variables). The stepwise multivariable model was adjusted for the following variables: age, sex, BMI, waist circumference, fasting glucose, fasting insulin, triglycerides, HDL cholesterol, and LDL cholesterol. R^2^ for the regression model was 0.479, 0.472 and 0.564 for the whole population, men and women groups respectively.

For the whole tested population, higher 25(OH)D was significantly associated with male sex, younger age and lower BMI. The relationship of 25(OH)D with several markers of metabolic disorder, such as waist circumference, fasting glucose, fasting insulin, triglyceride, HDL cholesterol and LDL cholesterol, remained significant in models adjusted for sex, age and BMI. For the sex specific multiple adjusted regression analysis, 25(OH)D was negatively related to age, BMI, WC, fasting plasma glucose, fasting insulin, triglyceride, and positively to HDL(P = 0.008) for male. And 25(OH)D was negatively related only to fasting plasma glucose, fasting insulin, triglyceride for female.

## Discussion

Findings from this study suggest that hypovitaminosis D is relatively common among young and middle-aged, northern Chinese individuals living in an urban environment, especially those who are obese and have metabolic syndrome. Additionally, we found vitamin D status was significantly associated with glucose homeostasis indices and lipid profile, which remained after adjustment for BMI.

This study consisted of a high risk population for metabolic syndrome, namely a young and middle aged, urban cohort who were office workers and physically inactive [14]. The prevalence of vitamin D deficiency and insufficiency was 28.6% and 37.6% respectively which is not as striking as the 69.2% and 24.4% for vitamin D deficiency and insufficiency respectively as previously reported in Chinese population by Lu et al. [18]. This deviation perhaps derived in part from the older age of participants in their study (50–70 years old) compared to the present study (35–60 years old). Additionally, the present study excluded patients who have overt diabetes, CVD and currently use anti-hypertensive medication and lipid drugs, which means participants in the present study have better metabolic conditions compared to Lu’s study. Although lower 25(OH)D levels were positively related to female sex in multivariable analysis, there was no significant difference in vitamin D statuses between males and females in our present study (data not shown). This may be attributed to the older age and higher BMI levels in the male group when compared to the female group, which may eliminate the advantage of male sex on vitamin D metabolism.

We also observed a greater prevalence of hypovitaminosis D amongst overweight/obese individuals. Even among lean individuals, lower vitamin D concentrations were associated with greater BMI, which is similar to results reported for other ethnic groups [19]. The observed association of low 25(OH)D with BMI may be due in part to the increased distribution volume of lipid soluble vitamin D to fat. There are also some experimental data that suggest vitamin D deficiency promotes lipogenesis through elevated parathyroid hormone [20] and could possibly modulate adipogenesis through vitamin D receptor-dependent inhibition of critical molecular components involved in differentiation and maturation of adipocytes [21]. Thus, increases in body fat mass could worsen the state of vitamin D deficiency, which may further increase body fat mass through vitamin D receptor regulation of pathways that are yet to be confirmed.

In this study, we observed significant associations of serum 25(OH)D with components of metabolic syndrome including waist circumference, triglyceride, HDL cholesterol and fasting glucose, but we did not find associations with blood pressure, even after adjusting for adiposity, which is in accordance with the findings from both the Cross-Sectional Study in the 1958 British Birth Cohort [7] and the NHANES III [22] study. Lu et al. [18] also noted that low 25(OH)D level increased risk for metabolic syndrome. Similar to result in Lu et al. study, the present study showed that the association between 25(OH)D and predictor variables were stronger in men than in women. However, results are conflicting as some previous investigations have only observed associations of 25(OH)D with anthropometric markers of metabolic syndrome (i.e. WC and BMI), but not with any of the clinical or serum parameters (i.e. fasting glucose, triglycerides, HDL cholesterol or blood pressure) [23]. Because excess weight is a major component of metabolic syndrome, the associations noted in our study could reflect an association of serum vitamin D with excess weight. Regardless of the associations with components of metabolic syndrome, we also found a significant inverse association for 25(OH)D with LDL-cholesterol, which was not included in Lu’s study. LDL-cholesterol is perhaps the most important risk factor of cardiovascular disease, and thus, this result underscores the importance of hypovitaminosis D as a correlate of cardiovascular disease as has been observed in other investigations [24].

Insulin resistance is considered a likely mechanism causing metabolic syndrome and has been implicated in increased cardiovascular disease [25]. In the present study, insulin resistance as determined by HOMA-IR was significantly greater in vitamin D deficient subjects. Although prior data are conflicting [26], some studies did observe an inverse association between concentrations of vitamin D and insulin resistance in different ethnic populations and age groups [27-29]. However, results from the Framingham Heart study found that adjusting for measures of central adiposity diminished the association between 25(OH)D and proxy measures of insulin sensitivity [19], implying that obesity may still the most important factor influencing the relationship between insulin sensitivity and vitamin D status. A study in an African-American cohort [30] suggested that vitamin D may have more influence on peripheral, rather than hepatic, insulin sensitivity, through the measurement of the OGTT-derived whole body insulin sensitive index (Matsuda index). Therefore, direct measures of insulin sensitivity, such as euglycemic clamp, frequently sampled intravenous glucose tolerance test (FSIGT,) or glucose tracer studies, will be needed to confirm results.

Several limitations of this study merit consideration. The results of our study may not be generalized to all racial/ethnic groups or age groups given that our sample was northern Chinese and young to middle-aged. Multivariable analyses were unable to adjust for the parathyroid hormone because this was not measured in our sample.

Notwithstanding the above limitations, the present study had several strengths. We used a work and lifestyle-based sample not selected on the basis of adiposity-related traits, cardiovascular disease risk factors, or vitamin D status. Our participants were young and middle aged, urban Chinese office workers, who were physically inactive with a high education level, traits indicating high risk for obesity and related metabolic disorders. All the participants lived in the same city for more than 5 years and blood samples were collected within two months (November and December), eliminating seasonal and geographic effects on the results. Our study excluded overt diabetes, CVD, hypertension and hyperlipidemia, which need medication to control, so this is a key population for primary prevention of type 2 diabetes and CVD. Intervention to this population would have significant impact on the control of chronic metabolic disease in a cost-effective manner.

Although there is mounting evidence linking vitamin D deficiency with obesity and related metabolic abnormalities, vitamin D intervention trials have had mixed results, which are likely due to different study populations, vitamin D replacement dosage, and intervention length. It is also possible that the link between vitamin D and cardiometabolic risk factors may reflect the fact that both vitamin D deficiency and metabolic disorders are prone to cluster in obese populations.

## Conclusions

25-hydroxyvitamin D deficiency is associated with obesity and related cardiometabolic risk factors in middle-aged, urban Chinese adults. In the future, randomized controlled trials are needed to establish a cause-effect relationship between vitamin D deficiency, obesity and its metabolic consequence and to evaluate the use the vitamin D3 in metabolic syndrome patients.

## Abbreviations

25(OH)D: 25-hydroxyvitamin D; BMI: Body mass index; HOMA-IR: homeostasis model assessment of insulin resistance; HDL: high-density lipoprotein; LDL: low-density lipoprotein.

## Competing interests

The authors declare that they have no competing interests.

## Authors’ contributions

XY designed the study, analysis and interpreted the data and draft the manuscript. XZ, YL collected the blood sample and carried out radioimmunoassay. SW and CS coordinated the trial and participate in enzymatic colorimetric assay. YC contributed to data acquisition and data analysis. QS conceived the study, involved in its design, coordination and helped to draft the manuscript. All authors have read and approved the final manuscript.

## References

[B1] Hypponen E Power C Vitamin D status and glucose homeostasis in the 1958 British birth cohort: the role of obesity Diabetes Care 2006 29 2244 2246 10.2337/dc06-0946 17003300

[B2] Konradsen S Ag H Lindberg F Hexeberg S Jorde R Serum 1,25-dihydroxy vitamin D is inversely associated with body mass index Eur J Nutr 2008 47 87 91 10.1007/s00394-008-0700-4 18320256

[B3] Martins D Wolf M Pan D Zadshir A Tareen N Thadhani R Felsenfeld A Levine B Mehrotra R Norris K Prevalence of cardiovascular risk factors and the serum levels of 25-hydroxyvitamin D in the United States: data from the Third National Health and Nutrition Examination Survey Arch Intern Med 2007 167 1159 1165 10.1001/archinte.167.11.1159 17563024

[B4] Yetley EA Assessing the vitamin D status of the US population Am J Clin Nutr 2008 88 558S 564S 18689402 10.1093/ajcn/88.2.558S

[B5] Forman JP Giovannucci E Holmes MD Bischoff-Ferrari HA Tworoger SS Willett WC Curhan GC Plasma 25-hydroxyvitamin D levels and risk of incident hypertension Hypertension 2007 49 1063 1069 10.1161/HYPERTENSIONAHA.107.087288 17372031

[B6] Schmitz KJ Skinner HG Bautista LE Fingerlin TE Langefeld CD Hicks PJ Haffner SM Bryer-Ash M Wagenknecht LE Bowden DW Association of 25-hydroxyvitamin D with blood pressure in predominantly 25-hydroxyvitamin D deficient Hispanic and African Americans Am J Hypertens 2009 22 867 870 10.1038/ajh.2009.88 19444222 PMC2865679

[B7] Hypponen E Boucher BJ Berry DJ Power C 25-hydroxyvitamin D, IGF-1, and metabolic syndrome at 45 years of age: a cross-sectional study in the 1958 British Birth Cohort Diabetes 2008 57 298 305 18003755 10.2337/db07-1122

[B8] Cheng S Massaro JM Fox CS Larson MG Keyes MJ McCabe EL Robins SJ O'Donnell CJ Hoffmann U Jacques PF Adiposity, Cardiometabolic Risk, and Vitamin D Status: The Framingham Heart Study Diabetes 2009 59 242 248 19833894 10.2337/db09-1011 PMC2797928

[B9] Lips P Vitamin D status and nutrition in Europe and Asia J Steroid Biochem Mol Biol 2007 103 620 625 10.1016/j.jsbmb.2006.12.076 17287117

[B10] Tseng M Giri V Bruner DW Giovannucci E Prevalence and correlates of vitamin D status in African American men BMC Public Health 2009 9 191 10.1186/1471-2458-9-191 19534831 PMC2708155

[B11] Armas LA Dowell S Akhter M Duthuluru S Huerter C Hollis BW Lund R Heaney RP Ultraviolet-B radiation increases serum 25-hydroxyvitamin D levels: the effect of UVB dose and skin color J Am Acad Dermatol 2007 57 588 593 10.1016/j.jaad.2007.03.004 17637484

[B12] Scragg R Sowers M Bell C Serum 25-hydroxyvitamin D, diabetes, and ethnicity in the Third National Health and Nutrition Examination Survey Diabetes Care 2004 27 2813 2818 10.2337/diacare.27.12.2813 15562190

[B13] Zhou BF Predictive values of body mass index and waist circumference for risk factors of certain related diseases in Chinese adults–study on optimal cut-off points of body mass index and waist circumference in Chinese adults Biomed Environ Sci 2002 15 83 96 12046553

[B14] Grundy SM Cleeman JI Daniels SR Donato KA Eckel RH Franklin BA Gordon DJ Krauss RM Savage PJ Smith SC Jr Diagnosis and management of the metabolic syndrome: an American Heart Association/National Heart, Lung, and Blood Institute Scientific Statement Circulation 2005 112 2735 2752 10.1161/CIRCULATIONAHA.105.169404 16157765

[B15] Holick MF Vitamin D status: measurement, interpretation, and clinical application Ann Epidemiol 2009 19 73 78 10.1016/j.annepidem.2007.12.001 18329892 PMC2665033

[B16] Bonora E Targher G Alberiche M Bonadonna RC Saggiani F Zenere MB Monauni T Muggeo M Homeostasis model assessment closely mirrors the glucose clamp technique in the assessment of insulin sensitivity: studies in subjects with various degrees of glucose tolerance and insulin sensitivity Diabetes Care 2000 23 57 63 10.2337/diacare.23.1.57 10857969

[B17] Chapuy MC Preziosi P Maamer M Arnaud S Galan P Hercberg S Meunier PJ Prevalence of vitamin D insufficiency in an adult normal population Osteoporos Int 1997 7 439 443 10.1007/s001980050030 9425501

[B18] Lu L Yu Z Pan A Hu FB Franco OH Li H Li X Yang X Chen Y Lin X Plasma 25-hydroxyvitamin D concentration and metabolic syndrome among middle-aged and elderly Chinese individuals Diabetes Care 2009 32 1278 1283 10.2337/dc09-0209 19366976 PMC2699709

[B19] Cheng S Massaro JM Fox CS Larson MG Keyes MJ McCabe EL Robins SJ O'Donnell CJ Hoffmann U Jacques PF Adiposity, cardiometabolic risk, and vitamin D status: the Framingham Heart Study Diabetes 2010 59 242 248 10.2337/db09-1011 19833894 PMC2797928

[B20] McCarty MF Thomas CA PTH excess may promote weight gain by impeding catecholamine-induced lipolysis-implications for the impact of calcium, vitamin D, and alcohol on body weight Med Hypotheses 2003 61 535 542 10.1016/S0306-9877(03)00227-5 14592784

[B21] Wood RJ Vitamin D and adipogenesis: new molecular insights Nutr Rev 2008 66 40 46 10.1111/j.1753-4887.2007.00004.x 18254883

[B22] Ford ES Ajani UA McGuire LC Liu S Concentrations of serum vitamin D and the metabolic syndrome among U.S. adults Diabetes Care 2005 28 1228 1230 10.2337/diacare.28.5.1228 15855599

[B23] McGill A-T Stewart JM Lithander FE Strik CM Poppitt SD Relationships of low serum vitamin D3 with anthropometry and markers of the metabolic syndrome and diabetes in overweight and obesity Nutrition journal 2008 7 4 10.1186/1475-2891-7-4 18226257 PMC2265738

[B24] Anderson JL May HT Horne BD Bair TL Hall NL Carlquist JF Lappe DL Muhlestein JB Relation of vitamin D deficiency to cardiovascular risk factors, disease status, and incident events in a general healthcare population Am J Cardiol 2010 106 963 968 10.1016/j.amjcard.2010.05.027 20854958

[B25] Reaven GM Insulin resistance, the insulin resistance syndrome, and cardiovascular disease Panminerva medica 2005 47 201 210 16489319

[B26] Tai K Need AG Horowitz M Chapman IM Vitamin D, glucose, insulin, and insulin sensitivity Nutrition 2008 24 279 285 10.1016/j.nut.2007.11.006 18187309

[B27] Pinelli NR Jaber LA Brown MB Herman WH Serum 25-hydroxy vitamin d and insulin resistance, metabolic syndrome, and glucose intolerance among Arab Americans Diabetes Care 2010 33 1373 1375 10.2337/dc09-2199 20332348 PMC2875457

[B28] Chonchol M Scragg R 25-Hydroxyvitamin D, insulin resistance, and kidney function in the Third National Health and Nutrition Examination Survey Kidney Int 2007 71 134 139 10.1038/sj.ki.5002002 17082756

[B29] Alemzadeh R Kichler J Babar G Calhoun M Hypovitaminosis D in obese children and adolescents: relationship with adiposity, insulin sensitivity, ethnicity, and season Metabolism 2008 57 183 191 10.1016/j.metabol.2007.08.023 18191047

[B30] Ashraf A Alvarez J Saenz K Gower B McCormick K Franklin F Threshold for effects of vitamin D deficiency on glucose metabolism in obese female African-American adolescents J Clin Endocrinol Metab 2009 94 3200 3206 10.1210/jc.2009-0445 19549742 PMC2819826

